# Body Mass Index Changes in Children and Adolescents Treated with Methylphenidate for Attention Deficit Hyperactivity Disorder

**DOI:** 10.22037/ijcn.v18i2.38134

**Published:** 2024-03-12

**Authors:** Maryam KOUSHA, Samaneh HASANPOUR Asli, Fatemeh ESLAMDOUST- SIAHESTALKHI, Yasmin SHOAR, Zohreh SHOAR

**Affiliations:** 1 Kavosh Cognitive Behavior Sciences and Addiction Research Center, Department of Psychiatry, School of Medicine, Guilan University of Medical Sciences, Rasht, Iran.; 2 Children’s Oncology Group (COG), Monrovia, CA, USA.; 3 Department of Pediatrics, Cottage Children’s Medical Center, Santa Barbara, CA, USA.

**Keywords:** Body Mass Index, Child, Adolescent, Attention Deficit-Hyperactivity Disorder, Methylphenidate.

## Abstract

**Objectives:**

Attention Deficit Hyperactivity Disorder (ADHD) and obesity are major pediatric public health problems. The present study aimed to examine the association between these two health parties in our pediatric populations.

**Materials & Methods:**

This study is a single group retrospective cohort study about Body Mass Index (BMI) changes in 149 children and adolescents between 3-18 years old with a diagnosis of ADHD based on one child and adolescent psychiatrist interview according to the Diagnostic and Statistical Manual of Mental Disorders 4th edition criteria (DSM-IV-TR). All participants were treated with methylphenidate. Besides, they were reassessed by the Kiddie Schedule for Affective Disorders and Schizophrenia-Present and Lifetime Persian version (K-SADS-PL-P). Furthermore, the height, weight, and BMI of participants were calculated. The data were analyzed by descriptive statistics, repeated measures, and Wilks’ lambda analysis using IBM SPSS Statistics version 23.

**Results:**

The mean age of patients was 8.2±2.6 years, and 71.8% were boys. The obtained results showed that those treated with methylphenidate for more extended periods had higher BMI increases (p <0.001). The change in BMI was not related to the age at the start of treatment (p = 0.125), but this index was significantly different based on the years under treatment (p = 0.002). Moreover, changes in BMI were not significant based on gender (p = 0.850), the type of ADHD specifiers (p= 0.686), and concomitant drugs (p = 0.783).

**Conclusion:**

This study’s findings suggest that long-term use of ADHD medications could raise the risk of obesity in children.

## Introduction

In recent decades, the risk of obesity and overweight in children and adolescents has been increasing worldwide, leading major public health problem (1, 2). The pathophysiology of weight gain is due to the interaction between genetic, environmental, and biological factors (1). Attention Deficit Hyperactivity Disorder (ADHD ranks among the most prevalent neurodevelopmental disorders in child psychiatry. It manifests as a consistent pattern of inattention and/or hyperactivity-impulsivity, disrupting the child’s daily functioning and development. (3). There have been accumulated studies looking into the pattern of weight gain and growth in children with ADHD. Additionally, many previous studies have investigated the effects of ADHD and its treatment with stimulants on decreasing appetite and poor weight gain and growth. Weight gain, obesity, and increased Body Mass Index (BMI), on the other hand, had been less of a focus in the past. Recent reports indicate that ADHD is one of the most prevalent conditions in obese/overweight individuals. The association between ADHD and overweight/obesity is well established (4-8). A literature review shows that in those with ADHD, the risk of being overweight and obese is higher than 50% and 40%, respectively (4). In a prospective follow-up study in boys with a history of childhood ADHD, the risk of obesity in adulthood was higher, implying a gender effect difference (9). Various hypotheses have been proposed to examine the association between ADHD and weight gain. ADHD increases the risk factors for obesity, ADHD and obesity in children have common risk factors, or obesity and its related comorbidities can mimic the clinical presentation of ADHD. Many studies have focused on the first hypothesis and the fact that the characteristics of impulsivity and concentration deficit, specifically lack of inhibitory control and poor executive function, can lead to inappropriate eating behaviors. Possible common maladaptive behaviors include impulsivity, overeating, and binge eating (10). Problems with planning and performance can also lead to failure to maintain a healthy diet (10-12). The role of common genetic risk factors between ADHD and overeating should not be underestimated. Patte et al. showed that the Dopamine receptors D2 and D4 are the underlying mechanism of food reward sensitivity or impulsivity/inhibitory control (13). Furthermore, low impulse control, difficulties in postponing gratification, decreased motor performance, distorted planning, and social rejections can all contribute to a lack of activity, a greater tendency to watch TV or play video games, snacking in front of the TV, and increased sedentary behaviors (14). Cortese (2019) focused on a complex bidirectional association between ADHD and obesity and described the underpinning factors, including genetic alterations, obesogenic environment, and behavioral factors. They reported the possible effects of paralimbic and fronto-limbic structures that control executive dysfunctions, the role of energy balance, sleep cycle alterations, and inflammatory mechanisms as underlying factors (5). Proposedly, the inattentive and impulsive behaviors of these children can be associated with dysregulated eating behaviors and a lack of motivation to participate in sports and physical activity (7). Studies have shown that overeating in the absence of hunger can contribute to the loss of control eating in ADHD (15). Additionally, the risk of obesity has been higher in those with comorbidities, primarily adjustment disorder and learning disabilities (16). The impact of medications for treating ADHD on obesity outcomes remains unclear. However, in a prospective cohort study of ninety children with ADHD on treatment with methylphenidate, our group showed a significantly higher BMI after 1-year treatment (17). To further assess the relationship between ADHD and obesity, this research conducted a single-group retrospective cohort study to investigate the BMI changes in children and adolescents with ADHD treated with methylphenidate, a central nervous system stimulant. 

## Materials & Methods

This retrospective cohort study was approved by the Ethical Committee of Guilan University of Medical Sciences (code: IR. GUMS. REC.1396.135). This study was conducted in an outpatient child and adolescent psychiatry clinic in Rasht, a city in northern Iran, in 2018. This clinic is a referral center for children and adolescents with various psychiatric disorders that covers all socioeconomic status and cultural groups of the community. There were 149 participants between the of ages three and 18 years who were previously diagnosed with ADHD by a child and adolescent psychiatrist according to the Diagnostic and Statistical Manual of Mental Disorders 4th edition criteria (DSM-IV-TR). Written informed consent and assent were obtained from the parents and their children whenever applicable. Eligible participants were reassessed using the Kiddie Schedule for Affective Disorders and Schizophrenia-Present and Lifetime Persian version (K-SADS-PL-P) to confirm the diagnosis, specifiers, and psychiatric comorbidities. This research excluded those who discontinued treatment and those with preexisting neurological and medical disorders, such​​​​​​​ as diabetes but not those with psychiatric comorbidities. Holiday and weekend breaks from medications were allowed. Baseline height and weight were extracted from patient files. During the observation period, all participants completed a medical history and physical examination, including anthropometric measures such as height, weight, and BMI. 

**Instruments**

K-SADS-PL-P: This is a semi-structured diagnostic interview tool to assess current, past, and lifetime diagnostic status in children and adolescents. The KSADS-PL-P has acceptable concurrent validity in diagnosing current significant disorders. The test-retest reliability of most of the current diagnoses was good to excellent. The sensitivity and specificity of the current diagnosis for ADHD are 90% and 91%, respectively (Kappa=0.8, p<0.000) (18).

Weight: measured in kilograms using a Seca weighing scale manufactured by a German company.

Height: measured in meters using a wall-mounted Seca stadiometer manufactured by a German company.

BMI: calculated by dividing weight in kilograms by the square of the height in meters (weight/height² in units of kg/m²). 

**Statistical analysis**

Data were analyzed using IBM SPSS Statistics version 23. This study used mean, standard deviation, frequency, and frequency percentage for descriptive statistics. In addition, the current study used repeated measures and Wilks’ lambda analysis to analyze the effect of multiple variables on BMI. A P value of less than 0.05 was considered statistically significant. 

## Results

Table 1 represents the demographic characteristics of patients with ADHD. One hundred and forty-nine patients with a mean age of 8.2±2.6 years enrolled in the study; 107 (71.8%) participants were boys. Those on treatment for more than six years (3 patients) were excluded from analyses due to the small sample size. Table 2 shows the BMI changes in those treated with methylphenidate.

Change in BMI and age of starting drug therapy

The age at the treatment’s start was insignificant (Wilks’ lambda statistic: 0.983, F = 2.378, with p = 0.125). However, the duration of treatment was significant (Wilks’ lambda statistic: 0.850, F= 3.540, with p = 0.002). 

Change in BMI and gender 

By entering the gender in repeated measures analysis, the effect of this variable on the BMI changes was investigated. This effect was removed from the model due to the lack of significance of the interaction between gender and the years under treatment (p = 0.411), and the model was re-fitted with constant effects. In the final model, the Wilks’ statistic for gender and years of treatment was calculated to be 0.814 and 1.0, respectively. The data showed that gender was not significant (p = 0.850), but the years of treatment were significant (p <0.0001).

Change in BMI and ADHD specifiers

The type of ADHD specifiers (Mixed, Inattentive, and Hyperactive-Impulsive) were included in the repeated measures analysis. The interaction between the treated years and the type of disorder was insignificant (p=0.688) and removed from the model. The model was fitted again with the main effects. The years under treatment (Wilks’ lambda= 0.791) were significant (p < 0.0001), but the type of disorder (Wilks’ lambda =0.995) was not (p= 0.686). Therefore, the obtained data indicated that types of ADHD disorder did not affect the changes in BMI.

Change in BMI and concomitant drug use

**Figure 1:**
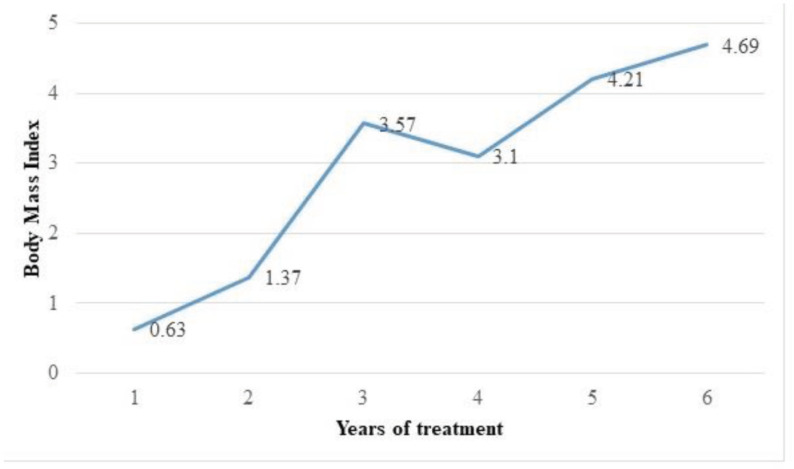
Mean BMI changes during years of ADHD treatment

The present study analyzed the effects of concomitant drugs (mood stabilizers, Serotonin Dopamine Antagonists, and Selective Serotonin Reuptake Inhibitors) by repeated measures analysis. The effect of this variable on the change in BMI was investigated. Due to the lack of significant interaction between the concomitant drugs and the years of treatment (p = 0.52), this effect was removed from the model and re-fitted. The effect of concomitant drugs on BMI change was not statistically significant (Wilks’ lambda statistic =0.996 and p = 0.783). In this model, the years of treatment were significant (p < 0.0001) (data not shown). Therefore, the results of this study revealed that the mean BMI change increased with treatment duration (Figure 1).

**Table 1:** Demographic characteristics of children with ADHD

	No. (%)
Gender	
Boys	107 (71.8)
Girls	42 (28.2)
ADHD Specifiers	
Mixed	122 (81.9)
Inattentive	6 (4)
Hyperactive/Impulsive	21 (14.1)
Comorbidities	
Anxiety Disorders	22 (14.8)
Autism Spectrum Disorders	16 (10.7)
Mood Disorders	7 (4.7)
Disruptive Behavioral Disorders	36 (24.2)
Without Comorbidities	68 (45.6)
Drug Treatment	
Only Methylphenidate	103 (69.1)
Methylphenidate and Mood Stabilizer or SDA	34 (22.8)
SSRIs	12 (8.1)

Abbreviations: SDA, Serotonin Dopamine Antagonist; SSRI, Selective Serotonin Reuptake Inhibitor.

**Table 2:** BMI changes during years of ADHD treatment

BMI (kg/m2)	Years of treatment					
	1 year	2 year	3 year	4 year	5 year	6 year
Number of patients	18	37	31	32	19	9
Baseline (mean± SD)	18.63±4.21	18.45±5.05	17.12±4.24	17.60±4.82	16.31±2.90	17.07±3.28
Follow up (mean± SD)	19.25±4.29	19.81±4.49	20.70±4.33	20.70±4.00	20.52±3.85	21.76±2.74
P-valuea	0.028	0.001	<0.0001	<0.0001	0.001	0.008

a repeated measure

## Discussion

The current study aimed to evaluate the changes in BMI in children and adolescents with ADHD treated with methylphenidate throughout one to six years, showing that children and adolescents treated with methylphenidate had gained more weight with increasing years of treatment. After adjusting for variables like gender, age at the start of treatment, ADHD subtypes, and prevalent psychiatric comorbidities, the length of methylphenidate treatment was the sole factor linked to weight gain and a rise in BMI. This research has revealed a consistent link between ADHD and obesity, aligning with earlier studies that have identified an increased risk of weight gain among individuals with ADHD. (4-8). In a cross-sectional community-based survey of 2,863 parents and their 11-17-year-old children, overweight/obese children were two times as likely to have an ADHD diagnosis (19). Hanson et al. proposed that binge eating disorder in ADHD was associated with increased BMI (11). Conversely, Holtkamp et al. showed that hyperactivity does not prevent obesity or overweight (20). 

Few studies pointed to the possible gender differences in the association between ADHD and weight gain (17, 21). Filers et al. concluded that ADHD in boys is a risk factor for being overweight, and the prevalence of overweight in girls with ADHD is age-dependent. Girls aged 10 to 12 have a 4-fold risk factor for obesity (21). Do et al. found that environmental factors affect the relationship between ADHD and obesity in boys, while genetic factors contributed to this relationship in girls (22). This research did not find similar gender differences.

In addition, this study did not find a significant association between ADHD specifiers, the age at the start of treatment, comorbidities, and the use of concomitant drugs in changes in BMI. In this study, only the treatment duration was a contributing factor in increasing BMI. This may indicate the abnormal and dysfunctional eating pattern of ADHD, specifically impulsivity in eating behavior, as the contributing factors (14). Similarly, the role of possible common genetic underpinnings cannot be ignored (5-7). 

Some studies have mentioned the role of physical inactivity in ADHD (23, 24). Kim et al. found that ADHD children, regardless of medication and gender, are less likely to engage in physical activity and organized sports (24). Hilbert et al. concluded that overeating in the absence of hunger might be associated with losing control of eating in ADHD children (15).

The obtained findings are inconsistent with previous studies that have shown that those with ADHD who are taking stimulant medications are less likely to be obese than those with ADHD who are not on medication (7). All children in this study were on medication, and the longer the duration of drug use, the higher mean BMI changes were observed. Schwartz et al. used longitudinal electronic health-related data on 16820 children and adolescents ages 3- 18. Researchers discovered that children with ADHD who were treated with stimulants experienced slower BMI growth in early childhood. However, these children later exhibited a rebound effect, resulting in BMI levels that surpassed those of children without ADHD or those not treated with stimulants. They concluded that stimulants appear to slow the rate of BMI growth in early childhood but later accelerate weight gain and growth rates in adolescence (10). In this study, all participants were treated with stimulants indicating the prominent role of behavioral factors and dysfunctional eating and activity patterns. Notably, parents often complain about impulsive eating behaviors after the effect of medication diminishes. Although it remains unclear if the duration of action of medication (short-acting versus long-acting stimulants) has any role in the observed behavior, this may point to the fact that a deficit in inhibitory control as a possible poor eating behavioral regulation can contribute to the risk of overweight and obesity in ADHD (17). Overall, the duration of treatment with methylphenidate had the most substantial impact on change in BMI in this study. 

The present study has some limitations and did not specify and stratify the results based on the dose of the methylphenidate. Additionally, this study did not include behavioral patterns associated with weight management, such as eating patterns, eating junk low-value foods, the number of meals, patterns of sleep and wakefulness, the hours of sitting in front of the TV, and the time spent in physical activities.

## In Conclusion

The current study implies a strong relationship between ADHD and obesity/overweight. This study’s results indicate that the duration of stimulant treatment in those with ADHD can contribute to the increased risk of obesity. Besides, the obtained results have substantial public health implications and, at a minimum, underline the importance of monitoring weight and BMI in patients with ADHD in practice settings and planning for behavioral preventive interventions. 

## Author’s Contribution

Conceptualization, investigation, and methodology. Samaneh Hasanpour Asli: Data collection. Yasmin Shoar and Fatemeh Eslamdoust-Siahestalkhi: formal analysis. Zohreh Shoar, Maryam Kousha and Fatemeh Eslamdoust-Siahestalkhi: Writing the original draft. Fatemeh Eslamdoust-Siahestalkhi and Maryam Kousha: writing-review & editing. Maryam Kousha: supervision

## Conflict of Interests

The authors declare that they have no conflict of interest.
